# ST-CRMF: Compensated Residual Matrix Factorization with Spatial-Temporal Regularization for Graph-Based Time Series Forecasting

**DOI:** 10.3390/s22155877

**Published:** 2022-08-05

**Authors:** Jinlong Li, Pan Wu, Ruonan Li, Yuzhuang Pian, Zilin Huang, Lunhui Xu, Xiaochen Li

**Affiliations:** 1 School of Civil Engineering and Transportation, South China University of Technology, Guangzhou 510641, China; 2 College of Computer Science and Technology, Harbin Institute of Technology, Shenzhen 518055, China; 3 Department of Civil and Environmental Engineering, University of Wisconsin-Madison, Madison, WI 53706, USA

**Keywords:** traffic time series forecasting, matrix factorization, residual learning, missing value

## Abstract

Despite the extensive efforts, accurate traffic time series forecasting remains challenging. By taking into account the non-linear nature of traffic in-depth, we propose a novel ST-CRMF model consisting of the Compensated Residual Matrix Factorization with Spatial-Temporal regularization for graph-based traffic time series forecasting. Our model inherits the benefits of MF and regularizer optimization and further carries out the compensatory modeling of the spatial-temporal correlations through a well-designed bi-directional residual structure. Of particular concern is that MF modeling and later residual learning share and synchronize iterative updates as equal training parameters, which considerably alleviates the error propagation problem that associates with rolling forecasting. Besides, most of the existing prediction models have neglected the difficult-to-avoid issue of missing traffic data; the ST-CRMF model can repair the possible missing value while fulfilling the forecasting tasks. After testing the effects of key parameters on model performance, the numerous experimental results confirm that our ST-CRMF model can efficiently capture the comprehensive spatial-temporal dependencies and significantly outperform those state-of-the-art models in the short-to-long terms (5-/15-/30-/60-min) traffic forecasting tasks on the open Seattle-Loop and METR-LA traffic datasets.

## 1. Introduction

Accompanied by the evolution of the urban intelligent transportation system (ITS), modern societies have benefited from a variety of human-centered traffic regulations. As one of the crucial steps for the ITS, searching for reliable and cost-effective traffic time series forecasting has been conducted for decades. The resulting advanced algorithms are vital for building ITS enabling cities to smarten up. In general, recorded data from multiple sensors form multivariate time series and can be interlinked [1]. Usually, the target of traffic time series forecasting involves the forward-looking estimation of traffic variables (e.g., volume, speed, or occupancy) and thus exploiting the potential for system efficiency upgrading and policy making. By the length of the time horizons, traffic forecasting can be briefly categorized into short-term (≤30 min) and mid- to long-terms (>30 min) [2]. Technically, traffic forecasting falls within the scope of multi-variate time series analysis, but it faces challenges due to the high complexity and dynamics of ITS [3]. Specifically, how to capture the inherent spatial-temporal correlations of traffic time series comprehensively becomes the primary challenge [2,4,5].

Against that background, a great deal of research effort has been carried out to address this issue. Broadly speaking, existing models for traffic forecasting can be roughly classified as classical models and deep learning (DL) models, the former of which can be further subdivided into statistical models and traditional machine learning (ML) models [6]. In detail, many earlier works simply concatenated a series of data from different locations into a vector and then applied the vector autoregressive (VAR) and autoregressive integrated moving average (ARIMA) and their variants for time series forecasting [7,8]. However, these types of statistical models face significant challenges from the ML models because it fails to take the spatial or temporal correlation into account [2]. As the representative of data-driven methods, the traditional ML models have also suffered from various difficulties in traffic forecasting. For example, the classical support vector regression (SVR) model takes the choices of kernel function and hyper-parameters very sensitively. And these models either impose ideal stationary assumptions or prevail a computational burden when dealing with large-scale traffic datasets [6]. Once confronted with overly complex historical data, they fail to account for the highly non-linear traffic properties.

Different from those previous attempts, the DL models have emerged as the predominant traffic prediction models owing to their thorough consideration of the factors affecting traffic conditions [9]. The paradigm of the models manifests itself as the stacking of basic learnable blocks, or layers, to create the specified deep architectures [10], and much progress has been made in related works. Among the modeling efforts, the class of methods like recurrent neural network (RNN) is famous for processing sequential data, and its variants of the long short-term memory network (LSTM) and the simplified gated recurrent network (GRN) are proven to be effective structures for resolving gradient vanishing of RNN in capturing the temporal dependencies of historical data [6,11]. Studies on real-world issues have verified the applicability and advantages of the LSTM or GRN in traffic forecasting tasks [10,12]. Unlike RNN-based temporal modeling, the graph-related models have shown a powerful impact on spatial feature learning of road networks [13]. Typically, the joint spatial-temporal graph convolutional network (STGCN) can efficiently raise the accuracy of traffic forecasting by modeling the multi-scale traffic networks [2]. Moreover, sequence-to-sequence algorithms as well as other technical tricks (e.g., attention and residual mechanisms) can also greatly augment the predictive accuracy [14,15]. Despite DL models performing well, the drawbacks are that the diversified spatial-temporal correlations have not been fully leveraged from traffic networks and excessive model training may worsen the overall prediction performance [2,16]. In contrast, matrix factorization (MF) dominates in traffic forecasting as it can rapidly capture the global spatial-temporal properties by a low-rank approximation relation. Furthermore, the regularization terms designed for factor matrices can also strengthen the modification of Matrix Factorization (MF) models [17,18]. Additionally, most existing studies mainly work on a historical time series assuming no missing data entries [3]. However, when collecting traffic data from ITS, data loss is inevitable due to the sensors or software failures [5,19], which makes it difficult to make accurate forecasts [20].

To solve the above problems, we have developed a spatial-temporal MF framework with compensatory residual modeling, which works together with GRN and graph Laplacian regularizers for traffic time series forecasting. Overall, the main contributions of this article are summarized as follows:

- We propose a novel forecasting model entitled ST-CRMF for depth extraction of the non-linear spatial-temporal dependencies within historical time series and its residuals, where the spatial-temporal regularizers and bi-directional residual structure greatly augment model performance.
- Apart from accurately predicting future traffic sequences, the ST-CRM can deal with incomplete traffic datasets and proves its predictive effectiveness in various missing cases.
- We empirically demonstrate the superiority of the ST-CRMF model on real-world traffic datasets (i.e., Seattle-Loop and METR-LA). The experimental results confirm that the proposed ST-CRMF model achieves satisfactory results for several pre-defined prediction lengths.

The rest of this article is organized as follows: Section 2 briefly introduces the existing literature; In Section 3, we illustrate the spatial-temporal matrix factorization and model architecture in detail; In Section 4, we describe the effectiveness of the ST-CRMF and its superiority over baseline methods, respectively; Finally, the concluding remarks are given in Section 5.

## 2. Related Work

Recently, traffic time series forecasting has attracted an increasing amount of research attention on account of the incentives of ITS [9]. Inspired by this, we first briefly review the forecasting of traffic time series under commonly encountered constraints, followed by their applications.

### 2.1. Traditional Multivariate Time Series Forecasting

Traditional multivariate time series forecasting mainly relies on a few mathematical and statistical modeling approaches [9]. In the early stages, Ahmed et al. [21] first proposed to apply the ARIMA model to calculate traffic data, and later Williams et al. [22] further used the seasonal ARIMA model to predict the short-term traffic flow. Furthermore, VAR extends the AR model to capture linear dependencies of multiple time series. For example, Yu et al. [23] introduced the AR regularizer for temporal modeling. Chen et al. [17] performed a matrix VAR for temporal regularization, which captured complex temporal dependencies as it utilized more parameters than AR. Nevertheless, the model complexity of these statistical methods grows quadratically with the number of variables [1]. Because of their capability of capturing non-linear relationships and dealing with high-dimensional time series, ML models have gradually outperformed the traditional models [6,9,11]. Since the re-activation of ML, including SVR, K-nearest neighbor (KNN), and random forest regression (RFR), are popularly employed for traffic time series forecasting thanks to their excellent learning capacity. Zhang et al. [24] built a hybrid forecasting framework based on SVR, RFR, and an enhanced genetic algorithm (GA) to improve the accuracy of traffic forecasting. Li et al. [25] developed an adapted SVR model for short-term traffic flow prediction. Besides, the hybrid models [8] combining statistical methods and ML have been successfully applied in traffic forecasting. Although these models exhibit certain advantages in terms of accuracy and interpretability of model parameters, they suffer from the constraints of large-scale traffic datasets and complex influences contained therein.

### 2.2. Deep Learning for Traffic Time Series Forecasting

Subsequently, DL has prevailed in correlated time series forecasting because of its superior ability to model complex functions and exploit underlying features without tricky feature engineering [26,27]. The capacity empowers traffic prediction to serve a vital role in ITS (e.g., driving decisions/services [28]). Previously, Lv et al. [29] confirmed that DL is effective for traffic prediction. Moreover, technically, DL models include those deep architectures formed by stacking several basic learnable blocks or layers and a series of related application paradigms have been developed and deployed. Ma et al. [30] first proposed a capsule network incorporating a nested LSTM for traffic state forecasting. Gu et al. [31] built the two-layer fusion DL (FDL) model combining LSTM and GRN to predict the traffic speeds of lanes. However, the potential of the RNN-based approaches is far from being adequately exploited. Except for sequential modeling, studies increasingly prefer to model historical data from temporal and spatial perspectives. As Zhao et al. [7] developed a temporal GCN (T-GCN) for capturing spatial and temporal dependencies. Fang et al. [32] proposed a meta-learning-based multi-source spatial-temporal network (Meta-MSNet) to model spatiotemporal characteristics of multi-source traffic data (e.g., weather, holiday notification). Graph WaveNet [4] integrated GCN with dilated causal convolution for saving the computational cost of dealing with long sequences. Moreover, Li et al. [33] built the dynamic graph convolutional recurrent network (DGCRN) which generated the dynamic adjacency matrices and extracted features from node attributes. Bai et al. [3] established the adaptive GCRN (AGCRN) for automatically capturing the node-specific spatial-temporal dependencies. Besides, like graph multi-attention network (GMAN) [34], they combined the attention mechanism with GCN to obtain more dynamic dependencies of traffic data.

### 2.3. Spatial-Temporal Modeling Using Incomplete Dataset

Most of the forecasting studies either delete incomplete data blocks or simply repair them via using interpolation models, but the removal of redundant data or unsatisfactory filling may induce a possible over-fitting [5]. A few models accomplished the prediction tasks along with data recovery. Cui et al. [10] developed the stacked bidirectional and unidirectional LSTM (SBU-LSTM) method with an imputation mechanism for network-wide traffic data repair and forecasting. However, this architecture inherently lacked the capability to extract spatial feature and so it failed to further enhance its overall performance. Matrix Factorization has been employed for traffic sequence recovery and prediction thanks to its approximation ability during the factorization process. Chen et al. [17] built a Bayesian temporal factorization (BTF) model by integrating a VAR layer into a Bayesian probabilistic matrix/tensor factorization algorithm to enable the forecasting and recovery of missing data. In the field of structural health monitoring (SHM), Ren et al. [35] established the incremental Bayesian matrix/tensor learning model for effective repair and response prediction of spatial-temporal data. Chen et al. [36] proposed a low-rank autoregressive tensor completion (LATC) framework, which introduced the temporal variation as the regularization term but overlooked the spatial factor. So far, despite the existence of a few models for time series recovery and prediction, the current methods still lack in-depth modeling of spatial-temporal data, especially for the residuals at the modeling stage. In this study, we propose a novel spatial-temporal factorization model ST-CRMF with residual learning for accurate forecasting and imputation of historical time series.

## 3. Methodology

### 3.1. Preliminaries

**Figure 1:**
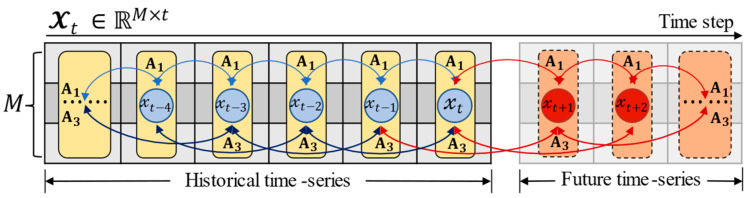
Illustration of multivariate time series forecasting (each ${𝔁}_{t}$ is a frame of traffic data at time step t).

In this study, we focus on formulating a nonlinear fitting function $f_{\theta }$ with partially historical series collected from a sensor network for traffic time series prediction over a certain time interval. One of the traffic variables can be speed, flow, and density, but it refers to traffic speed in our experiment. We first describe the symbolic representation of variables and relevant concepts in detail and then give a formal problem definition. Figure 1 shows the illustration of multivariate time series forecasting.

**Definition** **1** **(Urban** **Sensor** **Network** **Topology).** *We assume that the sensor network is divided as a 2D grid map of the size *𝓂×𝓃 *by its longitude and latitude, each of which is an equal-sized cell region. Then, the topology of this sensor network is defined as an unweighted graph expressed by* G=(V,ℰ) *. Where* $V\in {\mathbb{R}}^{M}$ *is a set of nodes and* M *is the number of nodes;* $ℰ\in {\mathbb{R}}^{N}$ *is a set of edges that represents the connectivity between road segments, and* N *is the number of edges.*

**Definition** **2** **(Traffic** **Network** **Matrix** **Sequence).** *As illustrated in Figure 1, the traffic speeds observed at the successive time intervals (*1, 2,⋯, t−1,t*) result in the matrix sequence*$\;X_{t}=\left\{{𝔁}_{1},{𝔁}_{2},\cdots ,{𝔁}_{t-1},{𝔁}_{t}\right\}$*, and* ${𝔁}_{ij}$ *stands for the traffic value on the*  ith  *node at the* jth *time slot,*  i∈{1, 2,⋯,M}*,*  j∈{1, 2,⋯,N}.

**Definition** **3** **(Problem** **Statement).**  *As per previous studies [37,38], the forecasting problem of traffic time series can be interpreted as learning the function*$f_{\theta \;}$ *based on the topology* G *and its feature matrix*  $X\in {\mathbb{R}}^{M\times N}\;$ *to predict the traffic speeds in the future period of* T  *with high accuracy. Therefore, we can formally define the studied problem in Equation (1) as follows:*

(1) $$\left\{{𝔁}_{t+T},{𝔁}_{t+T-1},\cdots ,{𝔁}_{t+2},{𝔁}_{t+1}\right\}=f_{\theta }\left(\left\{{𝔁}_{t},{𝔁}_{t-1},\cdots ,{𝔁}_{2},{𝔁}_{1}\right\};G\right)$$

*where* $\;f_{\theta \;}\left(\cdot \right)\;$ *denotes the desired nonlinear mapping function;* θ *represents the learnable parameters;* T *refers to the length of traffic time series needed to be predicted.*

### 3.2. Spatial-Temporal Matrix Factorization

#### 3.2.1. Matrix Factorization Description

**Figure 2:**
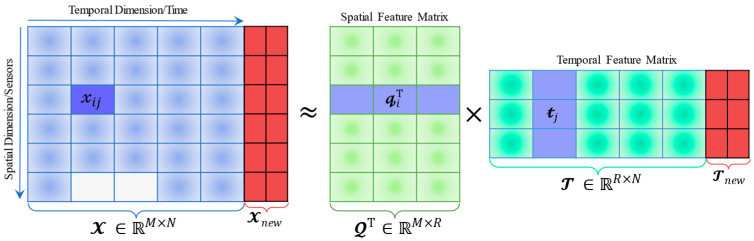
Schematic of the spatial-temporal matrix factorization (white boxes denote missing data).

In general, multivariate traffic time series involves both spatial and temporal dimensions, and our forecasting task revolves around modeling the implied spatial-temporal features to establish a study paradigm [9]. Following the general framework of available MF models [5,17], the standard MF as depicted in Figure 2 satisfies the basic process of Equation (2) as follows:

(2) $$X\approx Q^{T}T;\;\mathrm{the}\;\mathrm{elementwise}\;\mathrm{is}\;{𝓍}_{ij}\approx {𝓺}_{i}^{T}{𝓽}_{j}$$

where $Q\in {\mathbb{R}}^{R\times M}\;$ is the spatial feature matrix whose row is $\;{𝓺}_{i}^{T}$; $T_{\;}\in {\mathbb{R}}^{R\times N\;}$ is the temporal feature matrix whose column is ${𝓽}_{j}$; their element-wise ${𝓍}_{ij}$ is estimated by the inner product of ${𝓺}_{i}^{T}$ and ${𝓽}_{j}$; R denotes the positive integer referring to matrix rank. In Figure 2, the future time series $X_{new\;}$ remains related with $T_{new\;}$ at the same time direction when completing the data approximation by Equation (2).

Naturally, the motivation behind modeling spatial-temporal traffic data aims to impose a low-rank structure on the above 2D matrix for capturing its dependencies within the framework of the low-rank factorization and finally reconstructing this approximate relationship by the product of $Q\in {\mathbb{R}}^{R\times M}\;$ and $T_{\;}\in {\mathbb{R}}^{R\times N\;}$ with R≪min{M,N}. More precisely, the dependencies can deliver valuable insights into the spatial-temporal structure and assist in traffic forecasting tasks [15]. Therefore, the general optimization problem of MF can be summarized as follows:

(3) $$\underset{\;Q^{*},\;\;T^{*}}{\min}\frac{1}{2}\underset{\left(i,j\right)\in \Phi }{\sum }{\left({𝓍}_{ij}-Q_{i}^{T}T_{j}\right)}^{2}+\frac{\lambda_{𝓆}\eta }{2}{\left\|Q\right\|}_{F}^{2}+\frac{\lambda_{𝓉}\eta }{2}{\left\|T\right\|}_{F}^{2}$$

where Φ represents the set of (i,j) pairs for which the matrix element $\;{𝓍}_{ij}$ is known (i.e., training set); $Q_{i}$ and $T_{j}$ stand for the  ith  row and jth  column vectors of the feature matrices Q and T, respectively; the factors of ${\left\|Q\right\|}_{F}^{2}$ and ${\left\|T\right\|}_{F}^{2}$ serve for the over-fitting prevention and stability enhancement and their coefficients $\lambda_{𝓆}$, $\lambda_{𝓉}$ and η adjust the degree of regularization. However, the traditional MF models lack an adequate consideration of the spatial-temporal dependencies, resulting in undesirable non-linear fits. As a result, we introduce the spatial-temporal regularizers in MF to reinforce their factorization process. Specifically, we employ the graph Laplacian [18] and GRN [12] as the spatial and temporal regularizers of MF to further characterize the spatial and temporal dependencies, respectively.

#### 3.2.2. Regularized Spatial-Temporal Matrices Modeling

Inherently, the adjacent sensor in road networks collects traffic data with similar characteristics and the degree of mutual influence varies with its urban environment (e.g., location). In graph-based models, one usually constructs similarity graphs by linking close neighboring data points in a feature space and processes them to strengthen the modeling effects [9]. With that in mind, graph Laplacian is a sensible choice as the spatial regularizer of MF. In theory, the graph Laplacian spatial regularizer penalizes the discrepancies between the spatial feature vectors and their adjacent neighbors. More specifically, for an unweighted graph G=(V,ℰ), the graph Laplacian matrix satisfies as follows:

(4) $$L_{G}=D_{G}-A_{G}$$

where $L_{G}$ is a positive semidefinite matrix; $D_{G}$ is the diagonal degree matrix of nodes V, of which each element on the diagonal satisfies $\;D_{G}\left(i,i\right)=\underset{k}{\sum }\;A_{G\;}\left(i,k\right)$, k∈{1,2,⋯,M}; $A_{G}$ is the adjacency matrix of G, which states whether there are adjacent edges between nodes.

Therefore, the graph Laplacian spatial regularizer can be formulated as follow:

(5) $$ℒ\left(Q|\theta \right)=tr\left(Q^{T}L_{G}Q\right)=\frac{1}{2}\sum_{i,k}^{M}\;A_{G\;}\left(i,k\right)\|{𝓆}_{i}-{𝓆}_{k}\|{}_{2}^{2}$$

where θ contains the learnable regularization parameters; tr(·) denotes the matrix trace operator.

**Figure 3:**
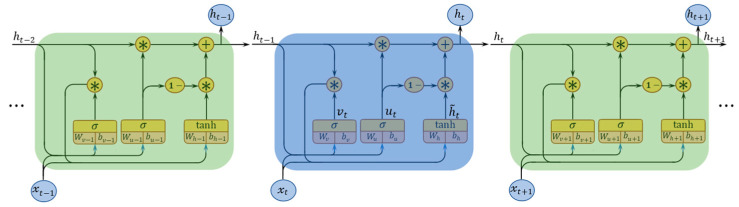
The structure of a GRN that consists of multiple GRU units.

To incorporate periodic temporal information, we apply GRN as the temporal regularizer of MF to capture multiple temporal dependencies. As depicted in Figure 3, GRN is a combination of the number of gated recurrent units (GRU), while GRU is a simplified variant of LSTM that uses two gate structures (i.e., reset and update gates). Both gates are the basis vectors that determine which information should be passed to the output. For each GRU cell, the sequential representation of the traffic status can be calculated iteratively by the following equations:

(6) $$\mathrm{Reset}\mathrm{Gate}:v_{t}=\sigma \left(W_{v\;}\left[{𝓍}_{t\;},h_{t-1\;}\right]+b_{v\;}\right)$$

(7) $$\mathrm{Update}\mathrm{Gate}:\;u_{t}=\sigma \left(W_{u\;}\left[{𝓍}_{t\;},h_{t-1\;}\right]+b_{u\;}\right)$$

(8) $$\mathrm{Cell}\mathrm{State}:\;{\tilde{h}}_{t}=tanh\left(W_{h\;}\left[{𝓍}_{t\;},v_{t}⊛h_{t-1\;}\right]+b_{h\;}\right)$$

(9) $$\mathrm{Output}:\;h_{t\;}=u_{t}⊛h_{t-1\;}+\left(1-u_{t}\right)⊛{\tilde{h}}_{t}$$

where $v_{t}$ and $u_{t}$ are the reset and update gates, separately; ($W_{v}$, $W_{u}$, $W_{h}$) and ($b_{v}$, $b_{u}$, $b_{h}$) stand for their weight and bias parameters; $h_{t-1}$, $h_{t}$, and $h_{t+1}$ are the previous, current, and next outputs; ${\tilde{h}}_{t}$ is the current cell state; ${𝓍}_{t-1}$, ${𝓍}_{t}$, and ${𝓍}_{t+1\;}$ are the previous, current, and next inputs; *tanh()* denotes the tanh activation function; The symbol “⊛” is the Hadamard product of the matrix. In this experiment, we use the sigmoid function (indicated by  σ) as the activation of the hidden state, and the hyperbolic tangent function (indicated by *tanh*) as the activation of output.

During temporal modeling, GRN offers a more efficient structure owing to fewer hyperparameters. And we adopt GRN as the temporal regularizer of MF, which can be formulated as follow:

(10) $$ℒ\left(T|\theta \right)=\frac{1}{2}\sum \|T-{\hat{T}\|}_{2}^{2}=\frac{1}{2}\sum_{j=\ell_{\delta }+1}^{N}\|{𝓽}_{j}-{𝓎}_{\theta }{\left({𝓽}_{j-\ell_{1}},{𝓽}_{j-\ell_{2}},\cdots ,{𝓽}_{j-\ell_{\delta }})\right\|}_{2}^{2}$$

where $\;{𝓎}_{\theta }\left(\cdot \right)\;$ is the GRN mapping function; $\hat{T}\;$ denotes the predicted T by GRN;$\;\varphi =\left\{\ell_{1},\ell_{2},\ell_{3},\cdots ,\ell_{\delta }\right\}$ stands for time lag set which represents the time-correlated topology.

Overall, by combining Equations (5)–(10), the objective function of the regularized MF is as follows:

(11) $$\underset{\;Q^{*},\;\;T^{*},\theta }{\min}Z\left(Q,T\right)=\underset{\;Q^{*},\;\;T^{*},\theta }{\min}\frac{1}{2}\underset{\left(i,j\right)\in \Phi }{\sum }{\left({𝓍}_{ij}-Q_{i}^{T}T_{j}\right)}^{2}+\frac{\lambda_{𝓆}\eta }{2}{\left\|Q\right\|}_{F}^{2}+\frac{\lambda_{𝓉}\eta }{2}\|{T\|}_{F}^{2}+\lambda_{𝓆}ℒ\left(Q|\theta \right)+\lambda_{𝓉}ℒ\left(T|\theta \right)$$

### 3.3. Overall Architecture of the ST-CRMF Model

**Figure 4:**
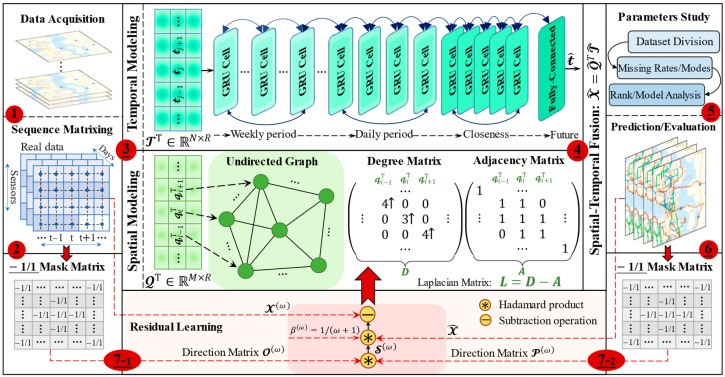
Architecture description and flow of the ST-CRMF model.

In this section, we will emphasize the overall architecture of the ST-CRMF model as detailed in Figure 4. Among them, the critical idea is to first model the spatiotemporal properties by the regularized MF model, then perform the complementary modeling of the resulting residuals to update MF model parameters, and finally predict the future time series precisely by the Equation (2) calculation. Besides, for the incomplete traffic datasets, we first repair the missing data and then conduct the forecasting tasks, but the latter is the focus of our study. Up next, we will describe the sampling and updating of spatial-temporal feature matrices to derive the model prediction and potential imputation.

#### 3.3.1. Model Implementation

Starting with the data acquisition and matriculation in Figure 4, the implementation of the ST-CRMF unfolds sequentially. In essence, the object of sampling and updating Q  and T  is to obtain a tractable closed-form of Equation (11) to enable the concrete expression of the spatial-temporal correlations between observations. As the closed-form solutions of Q and T  involve mutually, it is difficult to perform the model inference by a single back propagation. Motivated by the work [17], our idea was to estimate the spatial-temporal feature matrices (i.e., Q and T) by utilizing an alternating minimization scheme. Specifically, we introduce a popular alternating least-squares (ALS) method to perform the inference on the regularized MF model. In our study, the ALS minimizes the cost function  Z(Q,T)  iteratively by optimizing each component individually while keeping other factor matrices fixed, then the issue can be reduced to a linear least-squares issue. Actually, the ALS is attractive for its simplicity and can also provide satisfactory results for low-rank MF models.

First, to update Q, we can write the partial derivative of Z(Q,T)  about Q as below:

(12) $$\frac{\partial ℒ\left(Q|\theta \right)}{\partial Q}=\frac{\partial tr\left(Q^{T}L_{G}Q\right)}{\partial Q}=L_{G}Q+L_{G}^{T}Q$$

(13) $$\frac{\partial Z}{\partial Q}=-\left(X-Q^{T}T\right)T+\lambda_{𝓆}\eta Q+\lambda_{𝓆}\left(L_{G}Q+L_{G}^{T}Q\right)$$

Let $\frac{\partial Z}{\partial Q}$ = 0, then we have Equation (14):

(14) $$Q={\left(TT^{T}+\lambda_{𝓆}\eta I+\lambda_{𝓆}\left(L_{G}+L_{G}^{T}\right)\right)}^{-1}XT$$

Next, to update T, the partial derivative of Z(Q,T) about T is given by:

(15) $$\frac{\partial Z}{\partial T}=-\left(X-Q^{T}T\right)Q^{T}+\lambda_{𝓉}\eta T+\lambda_{𝓉}\left(T-\hat{T}\right)$$

Let $\frac{\partial Z}{\partial T}$ = 0, then we have the Equation (16):

(16) $$T={\left(QQ^{T}+\lambda_{𝓉}\eta I+\lambda_{𝓉}I\right)}^{-1}\left(XQ+\lambda_{𝓉}\hat{T}\right)$$

Additionally, we update the weights and biases of the GRN regularizer by a back-propagation with batch gradient descent until it satisfies that the error value of Z(Q,T)  falls below a set criterion. Then, we make rolling forecasting for future temporal feature vector ${\hat{𝓉}}_{j+1}$ in GRN regularizer by Equation (17)

(17) $${\hat{𝓉}}_{j+1}={𝓎}_{\theta }\left({𝓉}_{j-\ell_{1}+1},{𝓉}_{j-\ell_{2}+1},\cdots ,{𝓉}_{j-\ell_{\delta }+1}\right)$$

In fact, the alternating scheme first updates the Q  and T through ALS and then updates GRN parameters by back-propagation in each iteration. Subsequently, we expand the temporal dimension of X iteratively by computing the $Q{\hat{𝓉}}_{j+1}$, and further update the parameters of MF model. Moreover, when faced with incomplete datasets, the product of Equation (2) can impute the missing data contained in  X  in parallel after completing the update of Q and T.

#### 3.3.2. Compensated Residual Learning

Although the above-trained traffic forecasting model can generate preliminary results, they lack sufficient competitiveness with state-of-the-art models [2,7] in terms of in-depth learning of spatial-temporal properties (e.g., daily and weekly periods in Figure 4). As such, we design a residual feedback structure that efficiently adjusts the training parameters of MF model, and thus strengthens the data processing capability of the model. In particular, the residual modeling operation shares the training parameters with MF model and further optimizes them iteratively by the well-designed bi-directional residual structure in Figure 4. As per Equation (1), the universal framework integrating both MF output and the compensated residual learning mechanism as follows:

(18) $$\left\{{𝓍}_{t+T}^{*},\;{𝓍}_{t+T-1}^{*},\;\cdots ,\;{𝓍}_{t+2}^{*},\;{𝓍}_{t+1}^{*}\right\}=f_{\hat{\theta }\;}\left(\left\{{𝓍}_{t},\;{𝓍}_{t-1},\;\cdots ,\;{𝓍}_{2},\;{𝓍}_{1}\right\},\;\;X_{res};\;G\right)$$

where $f_{\hat{\theta }\;}\left(\cdot \right)$ represents the well-retrained MF model parameterized by G and residual matrix $X_{res}$; $\hat{\theta }$ denotes the modified training parameters; $X_{res}$ denote the residuals between the predicted values $\hat{X}=\left\{{\hat{x}}_{t},\;{\hat{x}}_{t-1},\cdots ,\;{\hat{x}}_{2},{\hat{\;x}}_{1}\right\}=f_{\theta }\left(\left\{x_{t},\;x_{t-1},\cdots ,\;x_{2},\;x_{1}\right\};\;G\right)$ and its ground truth values.

As interpreted in Figure 4 for the architecture design, our residual computational unit includes the feature matrix  X  and its direction matrix  O, the prediction matrix $\hat{\;X\;}$ and its direction matrix  P, the residual matrix $\;X_{res\;}$ and the bi-directional matrix  S. Of these, for the matrices  O  and  P, the elements of ${ℴ}_{ij}$ and ${𝓅}_{ij}$ both satisfy the categorical criteria as follows:

(19) $$\begin{array}{c} \mathrm{if}\;\mathrm{the}\;{𝓍}_{ij}/{\hat{𝓍}}_{ij}>0,\mathrm{the}\;{ℴ}_{ij}/{𝓅}_{ij}=1\\ \mathrm{if}\;\mathrm{the}\;{𝓍}_{ij}∕{\hat{𝓍}}_{ij}\leq 0,\;\mathrm{the}\;{ℴ}_{ij}∕{𝓅}_{ij}=-1 \end{array}$$

Next, we calculate the Hadamard product of the matrices O and  P to generate the above S, whose bi-directional attributes assist the MF model in acquiring more information within residuals. With these prerequisites ready, the single computation procedure of residual learning is as below:

(20) $$X-\beta \left(S⊛\left(Q^{T}T\right)\right)=X_{res},\;\;\beta =\frac{1}{\omega +1}$$

where ω is the training number for both MF modeling and residual learning; β is the step coefficient, which varies with the number of iterations. At each iteration, of particular interest is that MF updates alternate with residual learning, which ensures that the residuals keep decreasing while the learning ability of the ST-CRMF model keeps enhancing. Additionally, in Figure 4, apart from the data processing (e.g., dataset division and missing data preparation), we perform the parameter analysis for multiple learnable variables to examine their effects on the performance of the ST-CRMF model.

### 3.4. Pseudo-Code of the ST-CRMF Model

The training procedure of the ST-CRMF model is summarized in Algorithm 1. Notably, we define time lag as the combination of different time intervals (e.g., φ={1, 3} in Figure 1) to account for a variety of periodical patterns (e.g., closeness in Figure 4). In particular, the selection of the number of iterations and ranks varies considerably and will be analyzed in detail in Section 4.3.
**Algorithm 1:** Training Procedure of ST-CRMF Model**Input:** Graph network G=(V,ℰ); Feature matrix X; Rank R; Missing rates/scenarios; Maximum iteration λ.
**Output:** Learned ST-CRMF model; Factor matrices Q,T  and forecasted/repaired $\hat{X}=Q^{T}T$; Future sequence.
**1.** Initialize all trainable parameters in ST-CRMF.
**2. For**
ω=0 **to** λ−1 **do**
**3.** Compute and update of ${𝓺}_{i}^{\left(\omega +1\right)}$ by the ALS solution in Equation (14):
$${𝓺}_{i}^{\left(\omega +1\right)}={\left(\underset{\left(i,j\right)\in \Phi }{\sum }{𝓽}_{j}^{\left(\omega \right)}{𝓽}_{j}^{\left(\omega \right)}{}^{T}+\lambda_{𝓺}\eta I+\lambda_{𝓺}\overset{M}{\underset{i,k}{\sum }}\;A_{G\;}\left(i,k\right)I\right)}^{-1}\left(\underset{\left(i,j\right)\in \Phi }{\sum }{𝓍}_{ij}{𝓽}_{j}^{\left(\omega \right)}+\lambda_{𝓺}\overset{M}{\underset{i,k}{\sum }}\;A_{G\;}\left(i,k\right){𝓺}_{k}^{\left(\omega \right)}\right)$$
**4.** Compute and update of ${𝓽}_{j}^{\left(\omega +1\right)}$ by the ALS solution in Equation (16):
$${𝓽}_{j}^{\left(\omega +1\right)}={\left(\underset{\left(i,j\right)\in \Phi }{\sum }{𝓺}_{i}^{\left(\omega +1\right)}{𝓺}_{i}^{\left(\omega +1\right)}{}^{T}+\lambda_{𝓽}\eta I+\lambda_{𝓽}I\right)}^{-1}\left(\underset{\left(i,j\right)\in \Phi }{\sum }{𝓍}_{ij}{𝓺}_{i}^{\left(\omega +1\right)}+\lambda_{𝓽}{\hat{𝓽}}_{j}^{\left(\omega +1\right)}\right)$$
**5.** Update the training parameters θ in GRN regularizer by back-propagation with batch gradient descent.
**6.** Compute the $\beta^{\left(\omega \right)}$ and the residual matrix $X_{res}^{\left(\omega +1\right)}$ by Equation (20), and have it replaced $X^{\left(\omega \right)}$:$$X^{\left(\omega \right)}-\beta^{\left(\omega \right)}\left(S^{\left(\omega \right)}⊛{\left(Q^{T}T\right)}^{\left(\omega \right)}\right)\;=X_{res}^{\left(\omega +1\right)}$$
**7.** Update the training parameters $\hat{\theta }$ according to their gradient and learning rate.
**8. Until** *met model stop criteria.*

**9.** Repair the possible missing values in X and then update it.
**10.** Rolling forecast of the future time series $\left\{{𝓍}_{t+T}^{*},{𝓍}_{t+T-1}^{*},\cdots ,{𝓍}_{t+2}^{*},{𝓍}_{t+1}^{*}\right\}$ by Equation (18).

## 4. Experiment Study

### 4.1. Datasets Description

**Figure 5:**
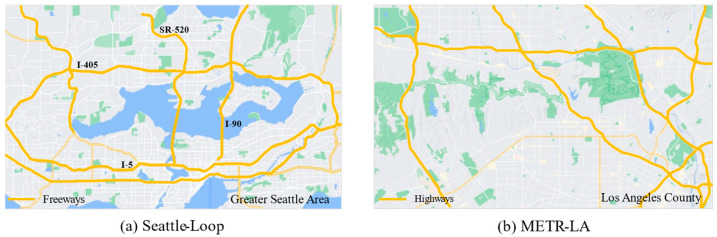
The area maps of the Seattle-Loop and METR-LA traffic speed datasets.

We conducted a lot of experiments on two publicly available and independent traffic speed datasets, i.e., Seattle-Loop and METR-LA for the training of ST-CRMF and its comparison with baseline models. Both datasets are widely used for traffic forecasting. The former was collected in 2015 on four connected freeways (I-5, I-405, I-90 and SR-520) in the Greater Seattle Area; the latter was collected on the highways of Los Angeles County from 1 March 2012 to 30 June 2012. Table 1 summarizes the key statistics [6,10] of both datasets, and Figure 5 provides their area maps [7,27]. In this experiment, we chose only the first two months of traffic data for modeling, and the sub-datasets were split in chronological order with 70% serving as the training set and the remaining 30% as the testing set.

### 4.2. Experimental Settings

#### 4.2.1. Baseline Models

To fully evaluate the predictive performance of ST-CRMF, we compared it with a series of baseline models under the same condition. Notice that we omitted the introduction of ARIMA, GRN, LSTM, and T-GCN models because of their simplicity and commonality.

(1) STGCN: *Spatio-Temporal Graph Convolutional Network* employs Chebyshev GCN and gated CNN for capturing the dynamics of spatial and temporal dependencies, respectively [2];
(2) AGCRN: *Adaptive Graph Convolutional Recurrent Network* captures the node-specific spatial and temporal correlations in traffic time series automatically without a pre-defined graph [3];
(3) Graph-WaveNet: *Graph WaveNet* integrates the diffusion graph convolutions with dilated casual convolution (called WaveNet) to capture the spatial-temporal dependencies simultaneously [4];
(4) PGCN: *Progressive Graph Convolutional Network* combines the gated activation unit and the dilated causal convolution to extract the temporal feature in traffic data [26];
(5) DGCRN: *Dynamic Graph Convolutional Recurrent Network* indicates that their dynamic graph can cooperate effectively with pre-defined graph while improving the prediction performance [33];
(6) GMAN: *Graph Multi-Attention Network* utilizes a variety of types of purely attention modules to learn complex spatial-temporal dependencies [34];
(7) GATs-GAN: The model incorporates *Graph Attention Networks* and *Generative Adversarial Network* to learn the node features and achieve the traffic state derivation [37];
(8) MRA-BGCN: *Multi-Range Attentive Bicomponent Graph Convolutional Network* uses the edge-wise graph construction, attention mechanism, and so on for traffic prediction [38].

#### 4.2.2. Measures of Model Effectiveness

We adopted four evaluation metrics to evaluate the effectiveness of the ST-CRMF: mean absolute percentage error (MAPE), root mean square error (RMSE) and mean absolute error (MAE), and the square of determination coefficient (i.e., $R^{2}$) [39,40]. The formulas for these indexes were defined as:

(21) $$ΜAPΕ=\frac{1}{\left|\Phi \right|}\underset{\left(i,j\right)\in \Phi }{\sum }\left|\frac{{𝓍}_{ij}-{\hat{𝓍}}_{ij}}{{𝓍}_{ij}}\right|\times 100$$

(22) $$\mathrm{RMSE}=\sqrt{\frac{1}{\left|\Phi \right|}\underset{\left(i,j\right)\in \Phi }{\sum }{\left({𝓍}_{ij}-{\hat{𝓍}}_{ij}\right)}^{2}}$$

(23) $$\mathrm{MAE}=\frac{1}{\left|\Phi \right|}\underset{\left(i,j\right)\in \Phi }{\sum }\left|{𝓍}_{ij}-{\hat{𝓍}}_{ij}\right|$$

(24) $$R^{2}=1-\frac{\sum_{\left(i,j\right)\in \Phi }{\left({𝓍}_{ij}-{\hat{𝓍}}_{ij}\right)}^{2}}{\sum_{\left(i,j\right)\in \Phi }{\left({𝓍}_{ij}-{\bar{𝓍}}_{ij}\right)}^{2}}$$

where |Φ| is the size of the index set Φ; ${𝓍}_{ij}\;$ and ${\hat{𝓍}}_{ij}\;$ are the actual and predicted values, respectively; ${\bar{𝓍}}_{ij\;}$ is the average of actual values. In general, a higher $R^{2}\;$ or lower other metrics will be a better model.

#### 4.2.3. Parameters Study

Recall that we aimed to learn the function $\;f_{\hat{\theta }\;}$ from historical data and then forecasting the next data. To better achieve such a desired goal, it was essential that we identified the best experimental parameters of the ST-CRMF through repeated trials. To be specific, the critical parameters of the ST-CRMF model include the size of GRN’s hidden cells, batch size, and the number of iterations. Considering both the performance and efficiency, the size of GRN’s hidden cells was set as rank R for both datasets and their batch size was set as 64. We trained ST-CRMF by Adam optimizer with an initial learning rate of 10^−4^ and utilized MAE as the loss function for GRN training. We used the Linear as the activation function of the fully-connected layer of GRN and apply a grid search with sliding window cross-validation to select the $\lambda_{𝓆}$,$\;\lambda_{𝓉}$ and η parameters, and employed the early stopping to avoid over-fitting. Besides, we set up a range of combinations of random missing (RM), cluster missing (CM), and hybrid missing (HM) scenarios [5] and 10–90% missing rates (by 10% steps) for ST-CRMF to test its repair effect.

### 4.3. Effect of Key Parameters on the ST-CRMF Model

**Figure 6:**
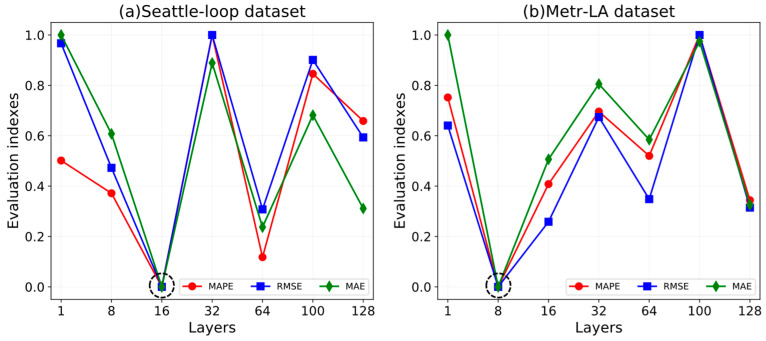
Impact of the hidden layers of GRN on the ST-CRMF model.

At first, the hidden layer of GRN exerted an influence on the prediction results of the ST-CRMF. For 5-min traffic forecasting, we undertook the parameters analysis by adding the hidden layers from 1 to 128. Figure 6 displays the parameter sensitivity of the ST-CRMF model with varying layer settings. We can observe that the ST-CRMF achieves better performance when the hidden layers of datasets (S) and (M) are set to 16 and 8, respectively.

**Figure 7:**
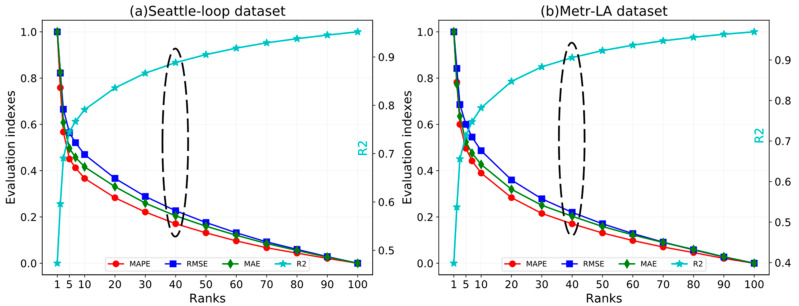
Impact of ranks on the evaluation metrics of the ST-CRMF model.

In traffic forecasting tasks, the choice of key parameters can define the performance frontiers of the ST-CRMF model. Thus, we further performed the parameter study affecting model complexity for investigating the effect of crucial parameters (e.g., ranks, the number of iterations) on the ST-CRMF. Note that all parameter test experiments of the ST-CRMF model were based on 5-min traffic forecasting. In each experiment, we altered the targeted parameter and kept other parameters fixed and Figure 7 shows the variation of the evaluation metrics of the ST-CRMF with different rank settings. Overall, the effect of ranks on the ST-CRMF complied with expectations. As rank increased, the metrics curves of both datasets (S) and (M) declined (MAPE, RMSE, and MAE) and ascend ($R^{2}$) significantly, and finally gradually stabilized. Theoretically, the optimal prediction results necessarily derive from the largest rank, but an excessive rank implies a greater consumption of storage and computational resource, and the trend of curves starts to slow down when the ranks exceed 40 in Figure 7. Hence, we finally ran the ST-CRMF model at the rank of 40 for both datasets from the balance of practical and theoretical.

**Figure 8:**
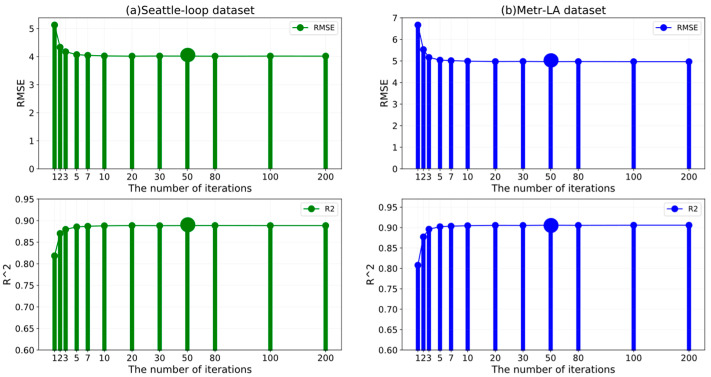
The number of iterations of the ST-CRMF model on the Seattle-Loop and METR-LA datasets.

One of the merits of the proposed ST-CRMF is the ability to perform compensatory modeling by means of designed feedback residual structure. Therefore, we further investigated the effect of training epochs on the efficiency of the ST-CRMF. Similarly, we altered the number of iterations while fixing all of the other parameters. As depicted in Figure 8, we showed the evaluation metrics of the ST-CRMF model on the datasets (S) and (M). With the increase in the training numbers, our ST-CRMF model did not suffer from the under-fitting problem owing to insufficient information. Instead, the RMSE and $R^{2}\;$ of the ST-CRMF model stabilized after about 50 iterations and the over-training may increase the complexity of ST-CRMF model with low return. Accordingly, all parameters were optimal values and should be identified by the dataset itself [41] and we determine 50 iterations for the datasets (S) and (M).

### 4.4. Empirical Results and Analysis

#### 4.4.1. Comparison with Baselines for 5-/15-/30-min Forecasting

As in most previous studies on fairness considerations, we provide some advanced baselines for comparison with the ST-CRMF. We adopted the default settings of the original proposals on the datasets (S) and (M) and listed some of the key parameters of the baseline models. Specifically, for Reference [7], the learning rate, batch size, and the training epoch of T-GCN were set to 10^−3^, 32, and 5000, respectively. For reference [37], the learning rates of generator and discriminator of GATs-GAN were 10^−3^ and 10^−5^, respectively. For reference [34], the number of attention blocks and heads were 3 and 8, respectively, and the dimensionality of each attention head was 8. For references [4,26], the PGCN and Graph-WaveNet models used the Adam optimizer with an initial learning rate of 10^−3^ for training. For reference [2], the ST-GCN executed a grid search to select optimal parameters. For reference [3], the dimension of the node embedding was one of the crucial parameters in AGCRN and its optimal value was 10. For reference [33], the layers, the size of the hidden state, and batch size of DGCRN were set to 1, 64, and 64, respectively.

Up next, the following tables present the evaluation metrics of the ST-CRMF and baseline models. Specific, Table 2 provides the prediction results on the dataset (M) for the 5-/15-/30-min forecasting.

Overall, the errors of all models increase with the prediction horizon, but the ST-CRMF achieves great performance over almost all-time periods, which verifies both the accuracy and stability of our model. At each time interval, our ST-CRMF outperforms (1) temporal models (e.g., LSTM, GRN, and T-GCN), confirming the importance of modeling spatial correlations; (2) spatial-temporal models (e.g., GMAN, PGCN, and Graph-WaveNet), demonstrating the benefits of compensated residual modeling. Further, the latest GATs-GAN model exhibits good performance, and even exceeds ST-CRMF at the first step, but it needs to improve its stability in the next intervals. Besides, we can derive another insight from Table 2 that the performance of all models tends to deteriorate accelerated with the multiplication of the forecasting period, and this will become increasingly visible in long-term forecasting.

#### 4.4.2. Comparison with Baselines for 15-/30-/60-min Forecasting

To further verify whether our model still maintains its superiority in long-term forecasting tasks, we investigated the results of ST-CRMF for 15-/30-/60-min forecasting on the METR-LA dataset. Table 3 presents the experimental metrics of all models. What stands out is that all metrics are almost consistent with the trend of the short-term forecasts in Table 2. One of the differences is that the trend of accelerated deterioration of the metrics in Table 3 becomes more apparent with time span, but it is in line with the law of traffic prediction. Besides, it is obvious that our ST-CRMF delivers competitive results under almost all evaluation metrics for all the forecasting periods. In particular, the ST-CRMF outperforms the classical forecasting models, such as ARIMA/STGCN by a large margin. Moreover, for the Graph-based models (e.g., DGCRN, MRA-BGCN), the ST-CRMF model performs much better thanks to its spatial-temporal regularizers and residual modeling. Hence, we can ascertain that our ST-CRMF enables complex traffic data prediction and satisfies both short-to-long terms forecasting tasks.

### 4.5. Ablation Study

**Figure 9:**
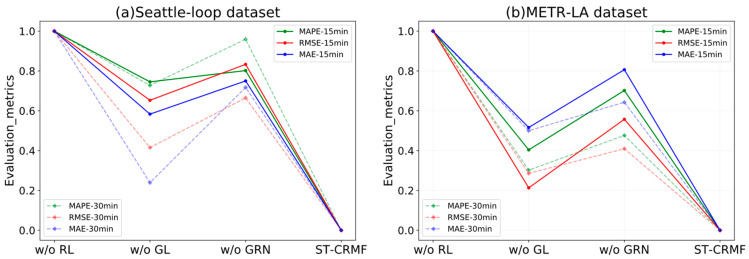
Ablation experiments of the ST-CRMF model on the Seattle-Loop and METR-LA datasets.

Similar to [2,5,27], we conducted a comprehensive ablation study on the Seattle-Loop and METR-LA datasets to investigate the effectiveness of key components that contribute to the improved results of the ST-CRMF model. We named the ST-CRMF without different components as follows: (1) ST-CRMF w/o GL: ST-CRMF without the graph Laplacian spatial regularizer; (2) ST-CRMF w/o GRN: ST-CRMF without the GRN temporal regularizer; (3) ST-CRMF w/o RL: ST-CRMF without the residual learning; and the baseline for the ablation study was the ST-CRMF model. Figure 9 shows the metrics of the ablation experiments for 15- and 30-min traffic forecasting tasks. We can conclude that: (1) compared with ST-CRMF model, all of the simplified ST-CRMF models will cause performance degradation; (2) both spatial and temporal regularizations of MF can improve its forecasting accuracy, but the latter is more effective; (3) the effect of bi-directional residual modeling is most evident as it further extracts more spatial-temporal information from residuals. All in all, this finding confirms that each component of the ST-CRMF model is indispensable for traffic time series forecasting.

### 4.6. Model Robustness Analysis

**Figure 10:**
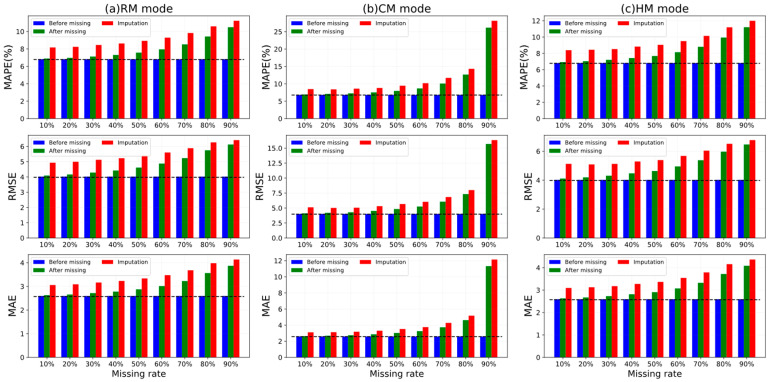
Imputation and prediction results of ST-CRMF model on Seattle-Loop dataset for RM, CM, and HM scenarios and 10–90% missing rates. Blue and green denote the predictive indicators of the non-missing and missing cases, respectively; red denotes the recovery metrics after missing.

As discussed in [5,19,36,42], there is often a data quality problem in real-world traffic time series. As such, we further investigated how our ST-CRMF performed under data loss, including its forecasting and recovery effects. We conducted several experiments on the combinations of RM, CM, and HM scenarios and 10–90% missing rates. But due to space constraints, we only present the experimental results of 5-min traffic prediction on the Seattle-Loop dataset. Note that we highlight the effect of data loss on the predicted results of the ST-CRMF model rather than on the comparison with repair models. Figure 10 shows the metrics of the ST-CRMF model for the repair and forecasting tasks in three cases.

In terms of prediction, we can observe that the evaluation metrics of the ST-CRMF increased under missing data relative to the non-missing case. More precisely, as the missing data rate increased, it satisfied the change that the forecasting accuracy of our ST-CRMF model remained at a high level but gradually followed an accelerated declining tendency. Specifically, for the extreme missing data rates exceeding 80%, the performance of the ST-CRMF model deteriorated so sharply that the forecasting tasks almost failed. Meanwhile, the different scenarios have different influences on the ST-CRMF. For the three scenarios, the MAPE, RMSE, and MAE of the ST-CRMF model varied most markedly under the CM mode due to its heaviest data structure corruption. Instead, the RM mode did impair the performance of the ST-CRMF model the least, which was in line with expected. Consequently, we should avoid the continuous loss of a large portion of time series during the acquisition phase. Besides, it can be found from Figure 10 that the repair results of ST-CRMF model followed its forecasting pattern when facing varied combinations of missing data rates and scenarios. Therefore, we can conclude that our ST-CRMF can deliver excellent prediction and repair results (except for extreme cases) under complex missing conditions.

### 4.7. Prediction Visualization

**Figure 11:**
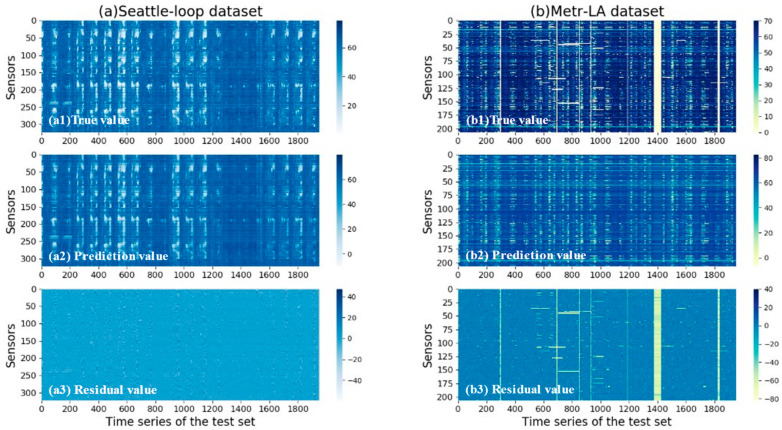
Visualization results of the predicted maps by the ST-CRMF model for 15-min forecasting interval.

We carried out a visualization survey for the ST-CRMF model to further validate its effectiveness. Figure 11 shows the heat maps of the ground and predicted values of ST-CRMF for 15-min forecasting. Of these, for datasets (S) and (M), which all exhibit the obvious periodic variations in Figure 11, our ST-CRMF model captured the periodicity better. Meanwhile, these residuals were very small overall, which also indirectly illustrates the superiority of the ST-CRMF model. In conclusion, both the above theoretical and visualization results indicate that our ST-CRMF model can effectively and accurately complete the tasks of forecasting and recovery of traffic time series.

## 5. Conclusions

The challenges of accurately predicting urban traffic time series arise from the extraction of non-linear spatial-temporal correlations. In this study, we proposed a novel matrix factorization framework (i.e., ST-CRMF) that integrated the spatial-temporal regularizers and compensatory residual learning to forecast/impute future/historical traffic time series, respectively. Overall, MF formed the core of our framework, which provided a research paradigm for subsequent complex spatial-temporal modeling. Here, we selected GRN as the temporal regularizer of MF to capture temporal correlations in time series; and graph Laplacian served as the spatial regularizer to exploit local spatial dependence information of adjacency sensors to strengthen the factorization process of MF. Further, we designed a bi-directional residual structure for compensatory modeling of the regularized MF by way of feedback optimization, which was in fact of great benefit to reinforcing the predictive performance of our ST-CRMF model.

Up next, the ST-CRMF shared and updated all the training parameters during rolling forecasting until it achieved the predetermined number of iterations. For each experiment, we performed the model inference on the ST-CRMF by an ALS method to estimate the spatial-temporal feature matrices of MF. We tested the effect of key parameters on the ST-CRMF through extensive experiments to identify their optimal values. Where for datasets (S) and (M), the hidden layers of GRN were set to 16 and 8 separately; the matrix ranks and the number of iterations were 40 and 50 respectively from the balance of practical and theoretical. Lastly, we evaluated ST-CRMF on two publicly available traffic speed datasets, Seattle-Loop and METR-LA. Experimental results indicated that our ST-CRMF outperformed other state-of-the-art baseline models (e.g., PGCN, GATs-GAN and DGCRN) under almost all evaluation metrics in the short- to long-terms (5-/15-/30-/60-min) traffic forecasting tasks, and the ablation experiments further confirmed the validity of key components of the ST-CRMF. Besides, our ST-CRMF can make predictions using the incomplete dataset while accomplishing data recovery. Except for extreme cases, our ST-CRMF model enabled highly precise imputation and prediction when facing the combinations of RM, CM, and HM scenarios and 10–90% missing rates.

In future studies, we will concentrate on improving the predictive efficiency through optimizing model parameters and increasing the forecasting accuracy by considering other factors (e.g., weather). Furthermore, we plan to test the proposed ST-CRMF model on more spatiotemporal traffic datasets.

## Figures and Tables

**Table 1:** Summary statistics of the Seattle-Loop and METR-LA traffic speed datasets.

	Datasets	Seattle-Loop (S)	METR-LA (M)
Information			
Location		The Greater Seattle Area	The Los Angeles County
No. of sensors		323	207
Time scope		1 January 2015~31 December 2015	1 May 2012~30 June 2012
Time granularity		5 min	5 min
Period step		288 (60/5 × 24)	288 (60/5 × 24)
Timestamps		105120	34272
Sources (https://github.com)		/zhiyongc/Seattle-Loop-Data (accessed on 2 July 2018)	/liyaguang/DCRNN (accessed on 2 October 2018)

**Table 2:** Prediction results of the ST-CRMF and baseline models on the Seattle-Loop dataset.

	Results	Dataset (S)								
		5-min			15-min			30-min		
Models		MAPE	RMSE	MAE	MAPE	RMSE	MAE	MAPE	RMSE	MAE
GRN [12]		8.74	4.92	3.24	9.67	5.39	3.48	10.23	5.76	3.63
LSTM [10]		8.17	4.70	3.09	8.88	5.15	3.28	9.80	5.67	3.55
T-GCN [7]		6.74	4.65	3.02	8.52	5.12	3.18	10.80	6.06	3.74
GMAN [34]		/	/	/	8.15	4.86	2.97	9.97	5.71	3.34
PGCN [26]		/	/	/	7.56	4.80	2.85	9.46	5.80	3.28
GATs-GAN [37]		**6.38**	**3.85**	2.65	7.63	4.56	2.97	8.89	5.19	3.46
Graph-WaveNet [4]		/	/	/	8.35	5.11	3.10	10.83	6.37	3.68
**ST-CRMF (Ours)**		6.81	4.02	**2.59**	**7.39**	**4.45**	**2.80**	**8.63**	**5.04**	**3.25**

Best results are highlighted in bold fonts and “/” stands for not available.

**Table 3:** Prediction results of the ST-CRMF and baseline models on the METR-LA dataset.

	Results	Dataset (M)								
		15-min			30-min			60-min		
Models		MAPE	RMSE	MAE	MAPE	RMSE	MAE	MAPE	RMSE	MAE
ARIMA [21]		9.60	8.21	3.99	12.70	10.45	5.15	17.40	13.23	6.90
STGCN [2]		7.62	5.74	2.88	9.57	7.24	3.47	12.70	9.40	4.59
AGCRN [3]		7.70	5.58	2.87	9.00	6.58	3.23	10.38	7.51	3.62
GMAN [34]		7.41	5.55	2.80	8.73	6.49	3.12	10.07	7.35	3.44
DGCRN [33]		6.63	**5.01**	2.62	8.02	6.05	2.99	9.73	7.19	3.44
MRA-BGCN [38]		6.80	5.12	2.67	8.30	6.17	3.06	10.00	7.30	3.49
Graph-WaveNet [4]		6.90	5.15	2.69	8.37	6.22	3.07	10.01	7.37	3.53
**ST-CRMF (Ours)**		**6.43**	5.05	**2.52**	**7.87**	**5.94**	**2.85**	**9.65**	**7.10**	**3.38**

## Data Availability

Not applicable.
